# Sporotrichosis in Domestic Cat and Zoonotic Transmission

**DOI:** 10.3201/eid3012.240864

**Published:** 2024-12

**Authors:** Sunil More, Timothy A. Snider, Akhilesh Ramachandran

**Affiliations:** Oklahoma State University College of Veterinary Medicine, Stillwater, Oklahoma, USA (S. More, A. Ramachandran); University of Missouri College of Veterinary Medicine, Columbia, Missouri, USA (T.A. Snider)

**Keywords:** sporotrichosis, fungi, differential diagnosis, zoonoses, One Health, Oklahoma, United States

**To the Editor:** Recent cases of feline sporotrichosis with zoonotic human infection have been highlighted in Kansas, USA (*1*). We describe a challenging feline sporotrichosis case in Oklahoma, USA, that emphasizes the critical need for early diagnostic strategies to mitigate the risk of further zoonotic transmission. Of note, the cat also scratched the veterinarian and owner and severe skin lesions subsequently developed on both of them; lesions resolved within 2 weeks (M. Carver, unpub. data, telephone report).

A 4-year-old domestic short-haired cat was taken for veterinary care with a raised, nodular, ulcerated mass on its right front foot. The mass was unresponsive to antibacterial treatment and progressively necrosed; the leg was subsequently amputated. A similar lesion developed on the left front foot. Skin biopsy samples from the cat’s left front foot were submitted for analysis. Histopathology revealed dermal infiltrates of neutrophils and macrophages with edema, fibrin, and karyorrhectic debris. We observed numerous intrahistiocytic and extracellular round to oval, faint basophilic 4-to 10-μm diameter yeasts surrounded by a clear halo (Figure, panel A, black arrowhead). Gram and Grocott methenamine silver stains showed yeasts with occasional cigar-shaped morphology (Figure, panel B, magenta arrow). We identified the fungal culture colonies as *Sporothrix schenckii* by using matrix-assisted laser desorption/ionization time-of-flight (MALDI-TOF) mass spectrometry. We cultured and identified a large number of secondary bacterial contaminants from the lesions. 

**Figure:**
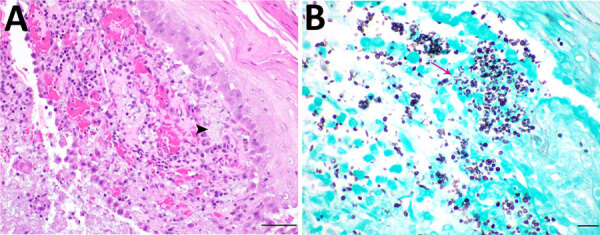
Skin lesions of sporotrichosis in a cat, Oklahoma, USA. A) Suppurative dermatitis with numerous 4-to 10-μm diameter intralesional yeasts (black arrowhead). Hematoxylin and eosin stain; scale bar indicates 50 μm. B) Occasional cigar-shaped morphology of *Sporothrix schenckii* (magenta arrow). Grocott methenamine silver stain; scale bar indicates 20 μm.

Sporotrichosis caused by dimorphic fungus of the genus *Sporothrix* presents potential diagnostic challenges because it can manifest in various clinical forms in human patients (*2*). The differential diagnoses for sporotrichosis in cats, because of overlapping clinical features, include feline leprosy, bartonellosis, atypical mycobacterial infections, *Staphylococcus* spp. pyoderma, dermatophytosis (ringworm), cutaneous lymphoma, deep mycoses (cryptococcosis, histoplasmosis, blastomycosis), and hypersensitivity reactions. Selecting appropriate diagnostic tests is crucial for accurately diagnosing sporotrichosis. Those multiple differential diagnoses emphasize the importance of incorporating histopathology, followed by fungal culture or PCR, for accurate diagnosis. To obtain reliable samples, deep punch or wedge biopsy specimens from intact nondraining lesions are recommended. Considering the zoonotic potential of *Sporothrix schenckii* infections (*1*,*3*–*5*), taking a One Health approach incorporating collaboration between veterinary and human healthcare sectors is essential for effective diagnosis and treatment of sporotrichosis cases. 
